# No genetic causal association between iron status and osteoporosis: A two-sample Mendelian randomization

**DOI:** 10.3389/fendo.2022.996244

**Published:** 2022-12-09

**Authors:** Jiawen Xu, Jun Ma, Jialei Chen, Shaoyun Zhang, Che Zheng, Haibo Si, Yuangang Wu, Yuan Liu, Mingyang Li, Limin Wu, Bin Shen

**Affiliations:** ^1^ Orthopedic Research Institute, Department of Orthopedics, Sichuan University West China Hospital, Chengdu, Sichuan, China; ^2^ Department of Orthopedics, The Third Hospital of Mianyang, Sichuan Mental Health Center, Mianyang, Sichuan, China

**Keywords:** osteoporosis, iron status, causal association, mendelian randomization, genetics

## Abstract

**Objective:**

To explore the genetic causal association between osteoporosis (OP) and iron status through Mendelian randomization (MR).

**Methods:**

Publicly available genome-wide association study (GWAS) summary data were used for MR analysis with four iron status-related indicators (ferritin, iron, total iron binding capacity, and transferrin saturation) as exposures and three different types of OP (OP, OP with pathological fracture, and postmenopausal OP with pathological fracture) as outcomes. The inverse-variance weighted (IVW) method was used to analyze the genetic causal association between the four indicators of iron status and OP. The heterogeneity of MR results was determined using IVW and MR–Egger methods. The pleiotropy of MR results was determined using MR–Egger regression. A leave-one-SNP-out test was performed to determine whether the MR results were affected by a single nucleotide polymorphism (SNP). The weighted median method was conducted to further validate our results.

**Results:**

Based on IVW, MR–Egger and weighted median models, we found no causal association between iron status (ferritin, iron, total iron binding capacity, or transferrin saturation) and OP (P_beta_ > 0.05 in all models). IVW and MR–Egger analysis of OP with pathological fracture and iron status indicators showed no potential genetic causal association (P_beta_> 0.05 in the two analyses). The results of the weighted median were consistent with those of IVW (P_beta_> 0.05 in all analyses). There was no potential genetic causal association between iron status and postmenopausal OP with pathological fracture based on serum iron (P_beta_>0.05 in all models). No heterogeneity or horizontal pleiotropy was found in any of the analyses. None of the leave-one-out tests in the analyses found any SNP that could affect the results of MR.

**Conclusion:**

Our results demonstrate that there is no genetic causal association between OP and iron status, but the effects of other factors were not excluded.

## Introduction

Osteoporosis (OP) is a systemic bone disease characterized by a reduction in bone mass and a deterioration of trabecular structure, resulting in decreased bone strength and an increased risk of fragility fractures (1). It is a chronic disease that exerts significant negative impacts on the health of elderly individuals, with a prevalence of 30-40% in women and a prevalence of 10-20% in men over 50 in mainland China (2). Established risk factors for OP include advanced age, endocrine disorders, malnutrition, obesity, and the use of drugs affecting bone metabolism (3). Moreover, some genetic mutations (three single nucleotide polymorphisms [SNPs] near the TNFRSF11B gene) contribute to the increased risk of OP (4). OP is occult and tends not to be diagnosed until the occurrence of fractures (commonly in the hip, caudal vertebrae, or forearm), which seriously compromise the life of patients. In Europe, the harm caused by OP complicated with fracture is second only to lung cancer (5). However, the current treatments of OP largely rely on medications such as bisphosphonates, calcitonin, oestrogen, and oestrogen receptor agonists, which have limited therapeutic effect and considerable adverse reactions (6). Therefore, further exploration of other risk factors for OP is imperative for the treatment and prevention of OP.

As an essential element for the human body, iron is indispensable for mitochondrial function, DNA synthesis and repair, and cell survival (7). These physiological activities acquire iron mainly through three pathways: the decomposition and destruction of ageing red blood cells by macrophages, absorption of iron in food to compensate for iron loss or increased demand, and the buffering effect of the iron reserve in the liver (8). Iron metabolism in the human body is mainly realized by regulating a series of iron metabolism-related proteins such as hepcidin, ferritin, transferrin, transferrin receptor, ferroportin 1 (FPN1), and divalent metal transporter 1 (DMT1). Problems in any one of these links lead to an imbalanced iron status in the body, resulting in a series of adverse effects (8). For example, iron deficiency can result in cognitive developmental defects in children and adverse pregnancy in women (9), whereas iron overload can damage multiple organs, including the liver, heart, and pancreas (10).

At present, the impact of iron status on OP remains unclear. Accumulating studies have revealed a certain correlation between iron status and OP at the biochemical level; i.e., an imbalance in iron status may increase the risk of OP (11–13). In contrast, some animal experiments have indicated that there is no correlation between iron status and OP (14). No final conclusion has yet been reached on the correlation between iron status and OP. Hence, it is necessary to conduct a more in-depth study on the correlation between iron status and OP at the gene level.

Mendelian randomization (MR) is an analytic approach to assessing the causal association between a modifiable exposure and a clinically relevant outcome (15). MR is based on Mendel's law of inheritance (parental alleles are randomly assigned to offspring) and uses genetic variants as instrumental variables (IVs), which can improve the limitations of some observational studies such as reverse causality, confounding factors, and various biases (16). Using genetic variation as an IV for exposure, MR studies can strengthen causal inferences about exposure-outcome associations by reducing confounding factors and reverse causality (17). To ensure the reliability of the results, MR must meet three assumptions: IVs are closely related to exposures, IVs are not related to other confounding factors, and IVs affect outcomes only through exposures and not through any other pathways (18). In recent years, accumulating evidence has demonstrated the reliability of two-sample MR analysis. For example, an existing MR analysis demonstrated that high iron levels have a positive causality with gout but a negative causality with rheumatoid arthritis (RA) (17). In addition, the genetic causal association between major depressive disorder (MDD) and osteoarthritis (OA) has also been documented in a recent MR study (19). However, few studies have focused on the association between iron status and OP using MR analysis. This study selected four serum biomarkers related to iron status (ferritin, iron, total iron binding capacity, and transferrin saturation) to explore the genetic causal association between iron status (exposure) and OP (outcome) through a two-sample MR analysis.

## Materials and methods

### Genome-wide association study summary data for iron status-related indicators

The genome-wide association study (GWAS) summary data of iron status-related indicators was obtained from a meta-analysis of three genome-wide association studies from Iceland, the UK and Denmark of blood levels of ferritin (N = 246,139), total iron binding capacity (N = 135,430), iron (N = 163,511) and transferrin saturation (N = 131,471). Information about the samples included in the study and data processing can be found in previous studies (20).

### Genome-wide association study summary data for osteoporosis

Recent large-scale GWASs and meta-analyses of OP in European populations (including OP N = 300,147, OP with pathological fracture N = 239,702, and postmenopausal OP with pathological fracture N = 173,601) were obtained from the FinnGen consortium. All three subtypes of OP were defined by the code M13 in the International Classification of Diseases, Tenth Revision (ICD-10). Detailed information on the participants, genotyping, imputation, and quality control can be found on the FinnGen website (https://www.finngen.fi/en) (21).

### Instrumental variable selection

We extracted genomic single-nucleotide polymorphisms (SNPs) associated with exposure (P<5×10^-8^). None of the instrumental SNPs were in linkage disequilibrium (LD). We performed the clumping process (R^2^ < 0.001, Magna window size = 10000 kb) to eliminate LD between the SNPs. The missing SNPs in the LD control group were also deleted. SNPs with a minor allele frequency (MAF) < 0.01 were removed. By default, if the SNP for a particular request does not exist in the resulting GWAS, the SNP (agent) with the requested SNP (target) in the LD is searched. The LD agent was defined using 1000 genomes of European sample data. In addition, to test whether there was a weak tool deviation in IV, we used the F statistic (F = R^2^ [n-k-1]/k [1-R^2^]), where R^2^ is the variance of exposure explained by selected instrumental variables (obtained from the MR Steiger directionality test), n is the sample size, and k is the total number of variables. If the F statistic of IV is much greater than 10, it indicates that the possibility of weak instrument variable bias is very small. In addition, we eliminated some IVs that may be related to confounding factors. These confounding factors included a lack of vitamin D and calcium, menopause and a lack of exercise.

### Mendelian randomization analysis

The “TwoSampleMR” package (version 0.5.6) of R language was used to analyze the genetic causal association between the four indicators of iron status and OP using three methods: inverse-variance weighted (IVW), MR–Egger, and weighted median (22).

The results were mainly based on IVW. The IVW method adopted a meta-analysis combined with Wald estimators from different SNPs. If each genetic variant can be used as an effective IV, the IVW method provides a consistent estimate of the causal effect of exposure on the outcome.

IVW and MR–Egger were performed to determine the heterogeneity of MR analysis results. Cochran’s Q statistic was adopted for IVW analysis, and Rucker’s Q statistic was used for MR–Egger analysis (23). The pleiotropy of MR results was detected using MR–Egger regression with an intercept P value > 0.05 indicating pleiotropy deficiency (24). A leave-one-SNP-out analysis was also conducted to investigate the possibility that the causal association between exposures and outcomes was driven by a single SNP (25). Moreover, we calculated the weighted median to further identify the potential causal association between exposures and outcomes.

Among the five methods of MR analysis, we mainly focus on the results of the IVW analysis. We considered that there was a genetic causal association between exposure and outcome when P<0.05 for the IVW analysis results. After correcting for multiple testing, the significance threshold of this study was P<4.17×10^-3^ (0.05/12=4.17×10^-3^). IVW and MR–Egger examined heterogeneity, and if P>0.05, there was no heterogeneity in our MR analysis. When our MR analysis results were free of heterogeneity/pleiotropy, we considered the IVW analysis results to be reliable.

## Results

After removing the SNPs of incompatible alleles, the details of all independent SNPs associated with exposure are shown in **Supplementary Table 1**. In our study, the F statistics of the instrumental variables associated with exposure were all greater than 10, indicating that the possibility of bias in weak instrumental variables was very small.

### The causal association between iron status and OP

Based on IVW and the MR–Egger model, we found that there was no causal association between iron status (ferritin, iron, total iron binding capacity, or transferrin saturation) and OP (P_beta_ > 0.05 in all models) (**Table 1**). In addition, the results of the weighted median showed that there was no potential causal association between OP and iron status indicators (P_beta_> 0.05 in all analyses) (**Table 1**).

**Table 1:** MR estimates from different methods of assessing the causal effect of.

Exposure	Outcome	NSNP	MR-Egger					Weighted Median					IVW				
			OR	(95%CI)	Beta	SE	P value	OR	(95%CI	Beta	SE	P value	OR	95%CI	Beta	SE	P value
serum ferritin	Osteoporosis	71	0.81	(0.52,1.26)	-0.20	0.22	0.36	0.88	(0.67,1.15)	-0.12	0.13	0.34	1.03	1.03(0.82,1.28)	0.02	0.11	0.82
	Osteoporosis fracture	71	1.42	(0.68,2.98)	0.35	0.37	0.35	1.34	(0.78,2.30)	0.29	0.27	0.28	1.41	1.41(0.97,2.04)	0.34	0.18	0.07
	Postmenopausal osteoporotic fracture	71	1.45	(0.60,3.54)	0.37	0.45	0.41	1.30	(0.72,2.33)	0.26	0.29	0.38	1.53	1.53(0.98,2.38)	0.42	0.22	0.06
serum iron	Osteoporosis	35	0.83	(0.64,1.09)	-0.18	0.13	0.19	0.83	(0.72,1.08)	-0.12	0.10	0.23	0.88	0.99(0.85,1.15)	-0.01	0.07	0.89
	Osteoporosis fracture	35	0.91	(0.44,1.87)	-0.09	0.36	0.83	0.91	(0.69,1.82)	0.11	0.24	0.66	1.12	1.06(0.71,1.57)	0.05	0.20	0.78
	Postmenopausal osteoporotic fracture	35	0.81	(0.35,1.91)	-0.20	0.43	0.65	1.13	(0.68,1.87)	0.12	0.25	0.64	1.15	1.15(0.71,1.85)	0.13	0.24	0.57
TIBC	Osteoporosis	53	0.93	(0.80,1.11)	-0.06	0.08	0.46	1.10	(0.94,1.28)	0.09	0.08	0.24	1.10	1.10(0.99,1.21)	0.09	0.05	0.07
	Osteoporosis fracture	53	0.74	(0.52,1.06)	-0.30	0.18	0.12	1.01	(0.74,1.36)	0.01	0.15	0.97	1.03	1.03(0.82,1.29)	0.02	0.11	0.81
	Postmenopausal osteoporotic fracture	53	0.77	(0.51,1.19)	-0.25	0.21	0.25	0.93	(0.66,1.32)	-0.06	0.17	0.71	1.02	1.02(0.79,1.33)	0.02	0.13	0.86
TSAT	Osteoporosis	51	1.02	(0.85,1.22)	0.02	0.09	0.83	1.03	(0.86,1.23)	0.02	0.09	0.78	0.96	0.96(0.85,1.08)	-0.04	0.06	0.48
	Osteoporosis fracture	51	1.34	(0.89,2.05)	0.29	0.21	0.17	1.16	(0.82,1.65)	0.14	0.17	0.41	1.12	1.12(0.86,1.47)	0.11	0.13	0.41
	Postmenopausal osteoporotic fracture	51	1.340	(0.81,2.21)	0.29	0.25	0.26	1.19	(0.81,1.76)	0.17	0.19	0.38	0.5	1.12(0.81,1.54)	0.11	0.16	0.50

MR, Mendelian randomization; IVW, inverse variance weighting; CI, confidence interval; TIBC, total iron-binding capacity; TSAT, transferrin saturation.

The MR–Egger intercept in the analysis showed that there was no horizontal multiplicity (MR–Egger intercept p value > 0.05) (**Table 2**). The MR–Egger test showed no heterogeneity in the MR analysis of iron status (ferritin, iron, total iron binding capacity, and transferrin saturation) and OP indicators (the Q-p values of the IVW and MR–Egger were both greater than 0.05) (**Table 3**). The leave-one-out analysis showed that the causal estimation of OP and iron status indicators was not driven by any single SNP (**Supplementary Figures S1–4**).

**Table 2:** MR-PRESSO analysis and MR-Egger intercept.

Exposure	Outcome	Egger-intercept	intercept-P value
serum ferritin	Osteoporosis	0.0099	0.2323
	Osteoporosis fracture	-0.0004	0.9747
	Postmenopausal osteoporotic fracture	0.0022	0.8963
serum iron	Osteoporosis	0.0133	0.1373
	Osteoporosis fracture	0.0114	0.6273
	Postmenopausal osteoporotic fracture	0.0260	0.3507
TIBC	Osteoporosis	0.0152	0.2182
	Osteoporosis fracture	0.0321	0.2614
	Postmenopausal osteoporotic fracture	0.0268	0.1124
TSAT	Osteoporosis	-0.0059	0.3871
	Osteoporosis fracture	-0.0179	0.2594
	Postmenopausal osteoporotic fracture	-0.0176	0.3524

MR, Mendelian randomization; TIBC, total iron-binding capacity; TSAT, transferrin saturation.

**Table 3:** Heterogeneity tests.

Exposure	Outcome	IVW		MR-Egger	
		Cochran’s Q	Q- P value	Cochran’s Q	Q- P value
serum ferritin	Osteoporosis	125.2424	0.1342	122.6614	0. 1125
	Osteoporosis fracture	82.6913	0.1424	82.6900	0.1246
	Postmenopausal osteoporotic fracture	96.7684	0.0188	96.7444	0.0754
serum iron	Osteoporosis	35.6018	0.3928	33.2644	0.4544
	Osteoporosis fracture	54.4280	0.0645	54.0347	0.2119
	Postmenopausal osteoporotic fracture	64.6602	0.1202	62.9508	0.0813
TIBC	Osteoporosis	47.8287	0.6386	42.2281	0.8043
	Osteoporosis fracture	61.8315	0.1651	56.0622	0.2908
	Postmenopausal osteoporotic fracture	68.5085	0.0621	65.1731	0.0876
TSAT	Osteoporosis	51.5270	0.4138	50.7383	0.4049
	Osteoporosis fracture	63.7456	0.0916	62.0955	0.0991
	Postmenopausal osteoporotic fracture	74.9640	0.0724	73.6395	0.0829

MR, Mendelian randomization; IVW, inverse variance weighting; TIBC, total iron-binding capacity; TSAT, transferrin saturation.

### MR analyses for OP with pathological fracture

IVW and MR–Egger analysis of OP with pathological fracture and iron status indicators showed no potential genetic causal association between the two (P_beta_> 0.05 in the two analyses) (**Table 1**). The results of the weighted median were consistent with those of IVW (P_beta_> 0.05 in all analyses) (**Table 1**).

In addition, no horizontal pleiotropy was found in this analysis (all intercept P values>0.05) (**Table 2**). The heterogeneity analysis found no heterogeneity in this analysis, with a Q-P value > 0.05 in the two analyses (**Table 3**). The results of the leave-one-out method in the initial analysis and verified analysis showed that no abnormal IV affected the overall results in the two analyses (**Supplementary Figures S5–8**).

### MR analyses for postmenopausal OP with pathological fracture

There was no potential genetic causal association between iron status and postmenopausal OP with pathological fracture based on serum iron (P_beta_>0.05 in all models) (**Table 1**). In addition, the results of the weighted median did not find a causal association between postmenopausal OP and pathological fracture and iron status indicators (P_beta_>0.05 in all analyses) (**Table 1**).

No horizontal multiplicity was found in this analysis (intercept P values in all analyses were greater than 0.05) (**Table 2**). The heterogeneity analysis showed no heterogeneity in the MR analysis of postmenopausal OP with pathological fracture and iron status indicators (Q-P value>0.05 in the IVW and MR–Egger analyses) (**Table 3**). The leave-one-out analysis showed that the causal estimation of postmenopausal OP with pathological fracture and iron status indicators was not driven by any single SNP (**Supplementary Figures S9–12**).

## Discussion

Studies have found that iron overload-induced ferroptosis in osteoblasts can inhibit osteogenesis and promote osteoporosis (26). Iron oxide nanoparticles (IONPs) can positively regulate bone metabolism *in vitro*, and daily administration of IONPs can alleviate oestrogen deficiency-induced osteoporosis by removing reactive oxygen species from the body (27). Although iron is strongly associated with OP, no studies have demonstrated a genetic causal association between iron status and OP. Studying the genetic causal association between iron status and OP is of considerable significance for research on the aetiology, mechanism and treatment of OP. This is the first study to explore the causality between iron status and OP through MR analysis. No evidence of positive or negative causality between iron status and OP was found, indicating that iron status had no genetic causality with OP. However, other factors (such as the environment) might also exert potential regulatory effects on the correlation between iron status and OP.

Iron status imbalance is manifested by iron overload or iron deficiency (28). Iron overload leads to an increase in serum iron, ferritin, and transferrin saturation but a decrease in transferrin, whereas iron deficiency shows the opposite trends (29). Previous studies have shown that these iron status-related indicators are related to OP or low bone mineral density (BMD). For example, the transferrin level in the serum of patients with osteoporotic hip fracture is lower than that of normal subjects (30). A cross-sectional study based on 4,000 women aged 12-49 found that serum ferritin is negatively correlated with BMD (31). Transferrin saturation shows a negative correlation with BMD in patients with transfusion-dependent beta-thalassemia (32). A controlled clinical trial revealed that the concentration of serum iron in OP patients is significantly higher than that in healthy controls, but it is believed that iron overload is a necessary but not sufficient condition for OP because iron deficiency may also affect OP (12). Moreover, a meta-analysis demonstrated a correlation between serum iron and OP and identified a low serum iron level as a risk factor for OP (33). Some scholars have proposed that both iron overload and iron deficiency may increase the risk of OP (13). High serum iron and ferritin levels may be beneficial to elderly individuals. A study based on 262 elderly rheumatoid arthritis (RA) patients found that the serum iron level of elderly RA patients was positively correlated with BMD (34). A study on elderly individuals aged 60 and above also revealed a positive correlation between serum ferritin and BMD (35). The association between iron status and OP obtained in previous population studies may be due to confounding factors such as sex and age (31, 35).

Notably, systemic iron overload is observed in hemochromatosis protein (HFE) knockout mice, but it does not have any effect on BMD and bone microstructure (14), which is consistent with our results that found no causal association between iron status and OP, at least at the genetic level. Although a polygenic risk score study found that ferritin is genetically correlated with systemic BMD (36), the sample was from the Caucasus (our sample is European), and the research method was not adequate to explain whether there is a causal association between the two. Hence, the rationality of our research results is still valid.

Iron overload-induced reactive oxygen species (ROS) can damage DNA, proteins, and lipids, eventually leading to cell death (37) and can also promote osteoclast differentiation and proliferation *via* the NF-κB signalling pathway (11). Furthermore, excessive iron inhibits osteoblast viability in a concentration-dependent manner. Mild iron deficiency facilitates osteoblast viability, whereas severe iron deficiency impedes osteoblast formation (38). As the main endogenous hormone regulating iron status, hepcidin can degrade ferroportin (FPN), the sole iron exporter on the cell membrane, resulting in an increase in intracellular iron levels (39). It should be noted that hypoxia and inflammation may affect the regulation of iron status by hepcidin (40), and OP is closely related to inflammation (41). Therefore, it is reasonable to speculate that some inflammatory factors affect the correlation between iron status and OP *via* hepcidin. Oestrogen affects iron status *via* hepcidin, and menopausal women often present with iron overload (42, 43). Moreover, oestrogen can also directly affect bone metabolism (44, 45). It is suggested that oestrogen may participate in the correlation between OP and iron status. In addition, some scholars have proposed the implication of environmental factors in the relationship between iron status and OP. A tendency towards OP (decreased BMD) has been found in some malnourished young people (45). It is well established that iron deficiency is a form of malnutrition, which suggests that a poor living environment with long-term malnutrition may be a common risk factor for iron status imbalance (long-term iron deficiency) and OP. To summarize, the association between OP and iron status is complex and dependent on multiple factors, but our results at least show that OP and iron status have no genetic causal association.

Observational epidemiological studies are prone to confounding factors, reverse causation and various biases and have generated findings that have proven to be unreliable indicators of causal effects (16). However, MR studies are free from the confounding factors (as in retrospective studies) and reverse causality of traditional epidemiological approaches (46). In addition, compared with observational epidemiological studies, MR analysis does not involve high measurement costs or a large number of appropriate biospecimens (16). Therefore, MR analysis has high reliability and is widely used in many studies (17, 47).

This study excludes a causal association between iron status and OP through MR analysis based on large-scale GWAS summary data, but it cannot be denied that iron status and OP may still be related. The MR method is not without limitations. First, MR analysis is heavily dependent on the reliable associations of genetic variants with the exposure(s) of interest, which are believed to have no effect on other phenotypes that might confound the association between the exposure and disease (48). In addition, the GWAS summary datasets used in this study were not stratified by population. The genotyping errors, phenotype misclassification, and confounding factors due to population stratification may cause spurious genetic associations, which will in fact be biased instruments for MR (15).

## Conclusion

This study shows that there are no positive or negative genetic causal associations between iron status and OP, but the influence of factors other than heredity cannot be ruled out.

## Data availability statement

The original contributions presented in the study are included in the article/**Supplementary Material**. Further inquiries can be directed to the corresponding author.

## Ethics statement

The source of the data was a publicly available database, and no human participants were involved; hence, ethical parameters are not applicable.

## Author contributions

Conception and design: JX, JM, JC, BS. Administrative support: JM, HS, BS. Provision of study materials: JX, JM, JC, CZ, YW. Collection and assembly of data: JX, LW. Data analysis and interpretation: JX, SZ, HS, YL, ML. Manuscript writing: JX, JC. Final approval of manuscript: All authors. All authors contributed to the article and approved the submitted version.
