# Supplementary material for: No genetic causal association between iron status and osteoporosis: A two-sample Mendelian randomization
Source: Front Endocrinol (Lausanne). 2022 Dec 9;13:996244. doi: 10.3389/fendo.2022.996244 (PMC9780364; doi:10.3389/fendo.2022.996244)

**Figure S1: Leave-one-out test plot of the causal association between serum ferritin and osteoporosis**


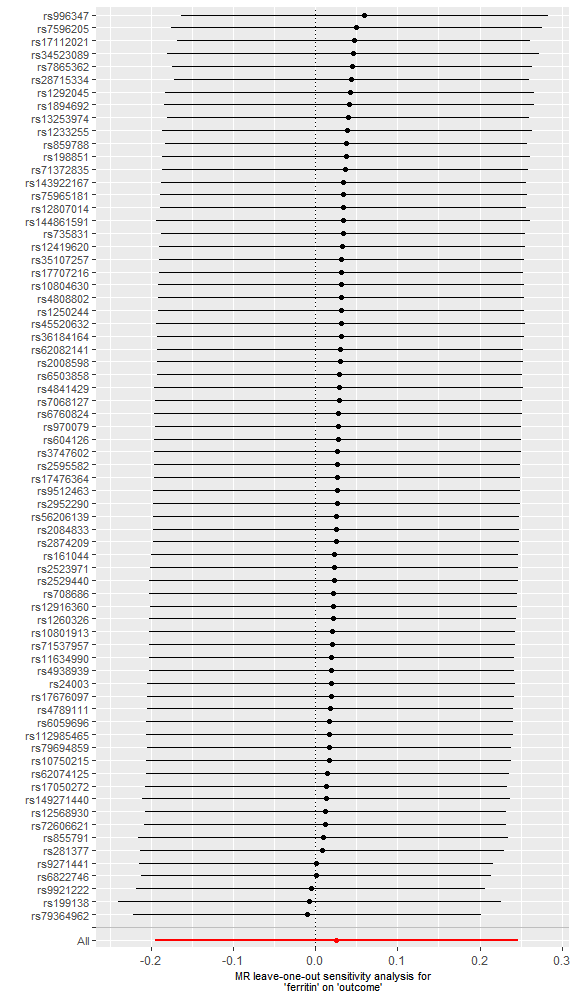


**Figure S2: Leave-one-out test plot of the causal association between serum iron and osteoporosis**


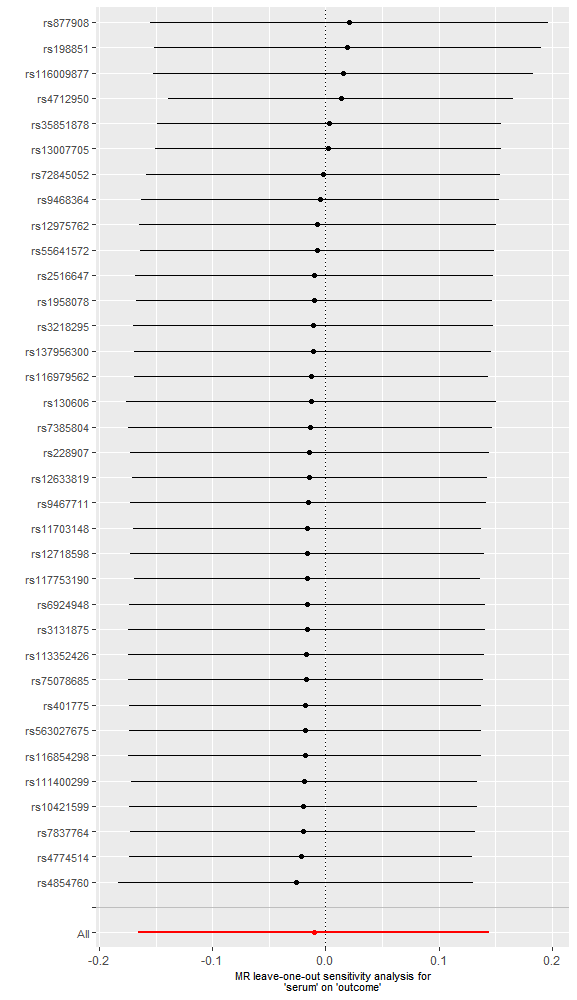


**Figure S3: Leave-one-out test plot of the causal association between total iron-binding capacity and osteoporosis**


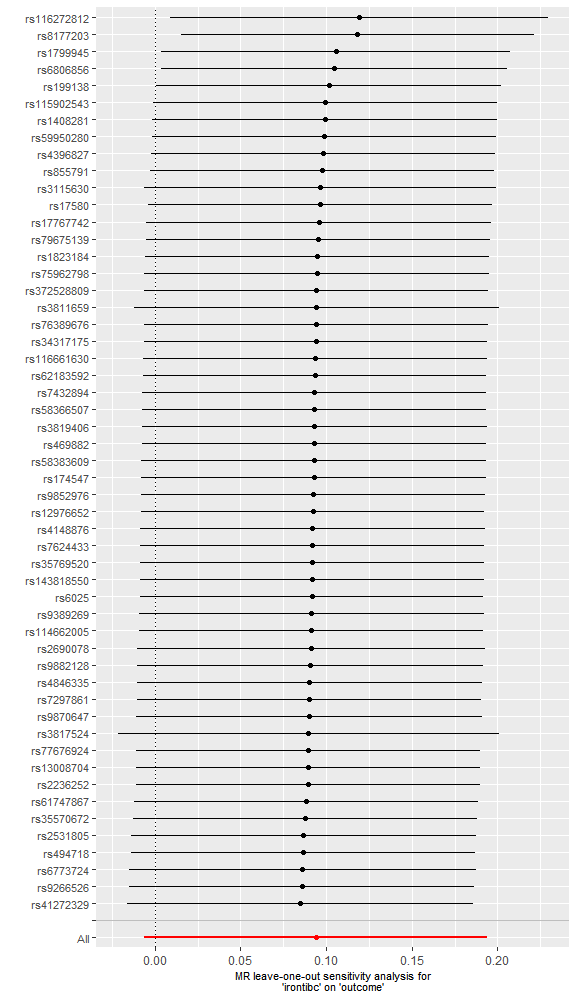


**Figure S4: Leave-one-out test plot of the causal association between transferrin saturation and osteoporosis**


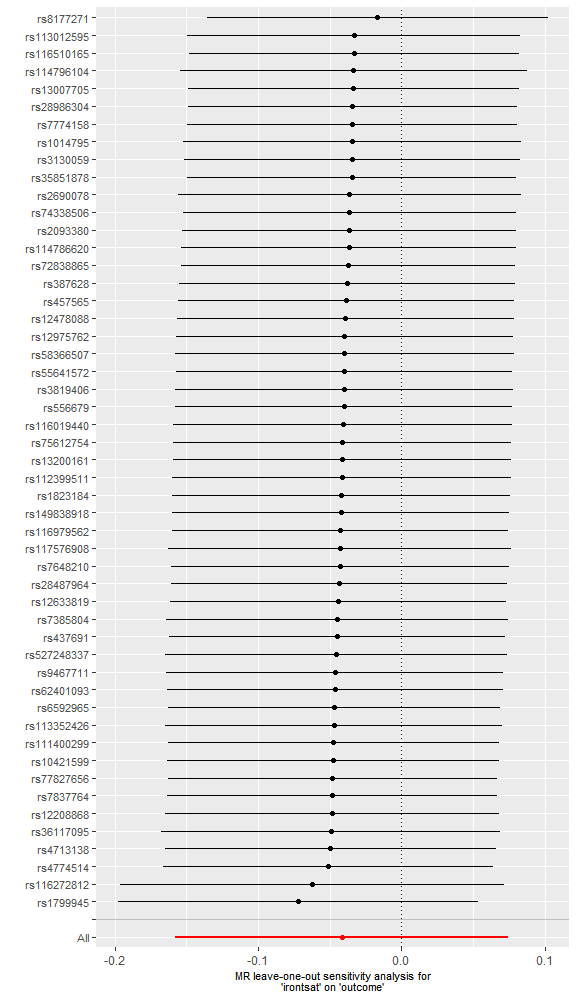


**Figure S5: Leave-one-out test plot of the causal association between serum ferritin and osteoporosis fracture**


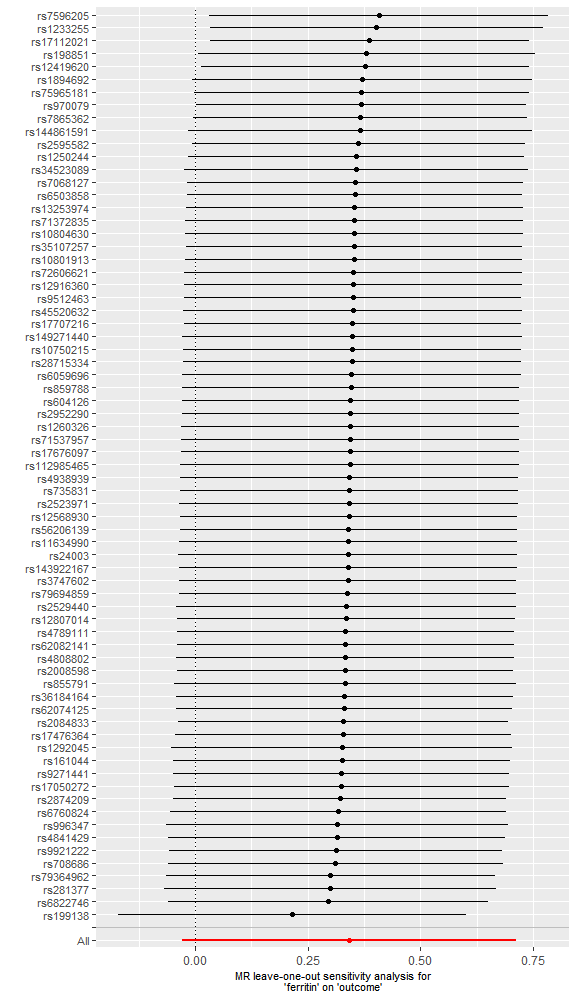


**Figure S6: Leave-one-out test plot of the causal association between serum iron and osteoporosis fracture**


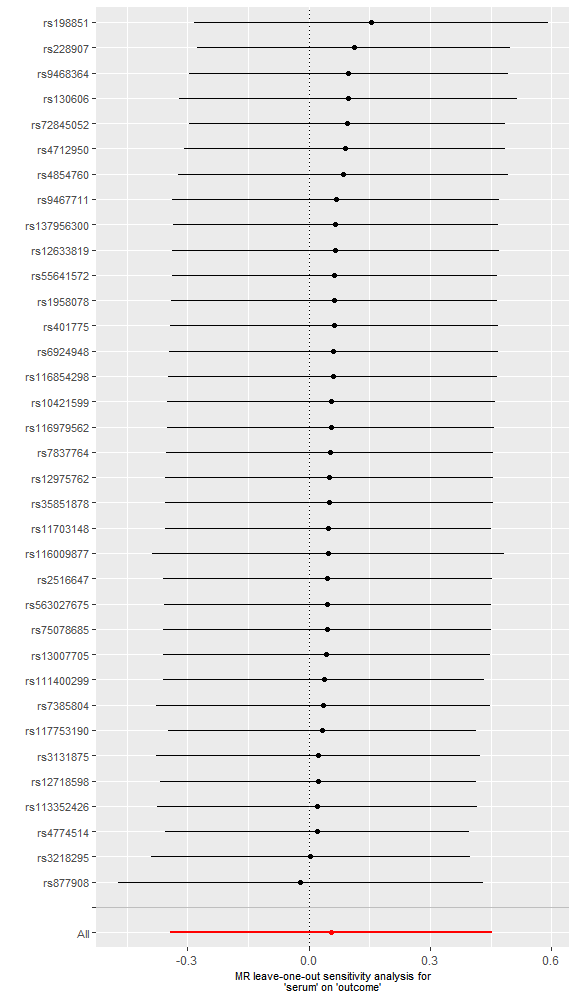


**Figure S7: Leave-one-out test plot of the causal association between total iron-binding capacity and osteoporosis fracture**


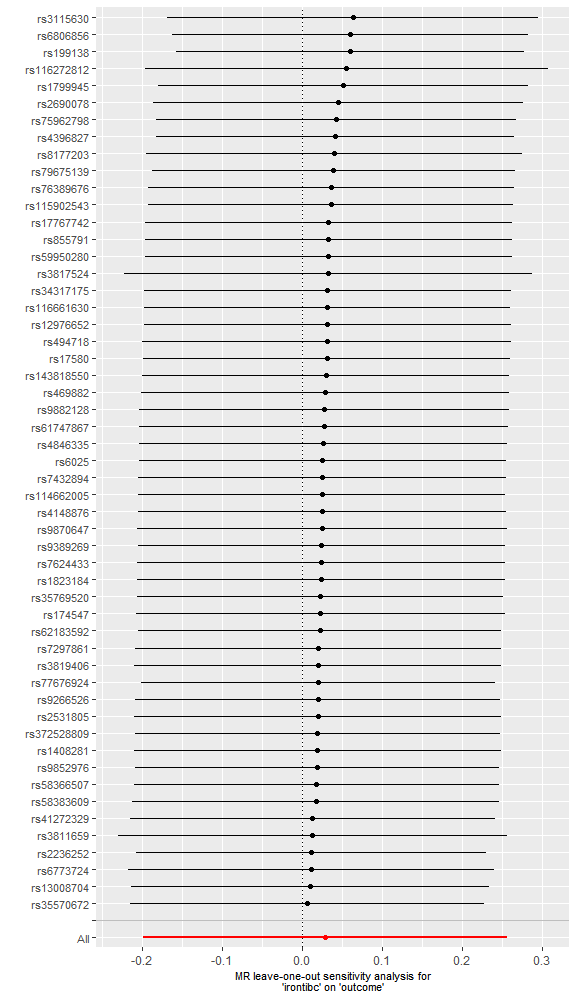


**Figure S8: Leave-one-out test plot of the causal association between transferrin saturation and osteoporosis fracture**


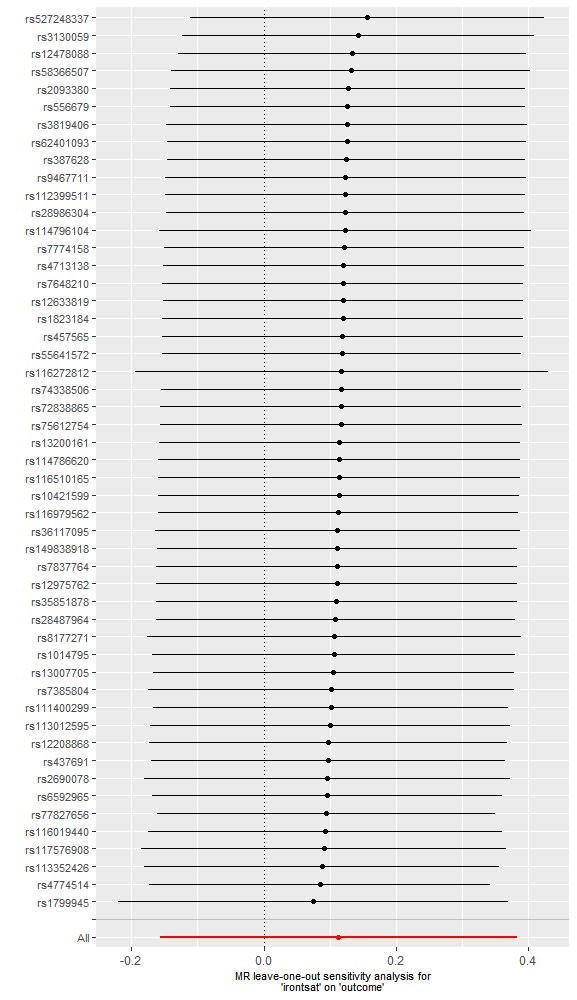


**Figure S9: Leave-one-out test plot of the causal association between serum ferritin and postmenopausal osteoporotic fracture**


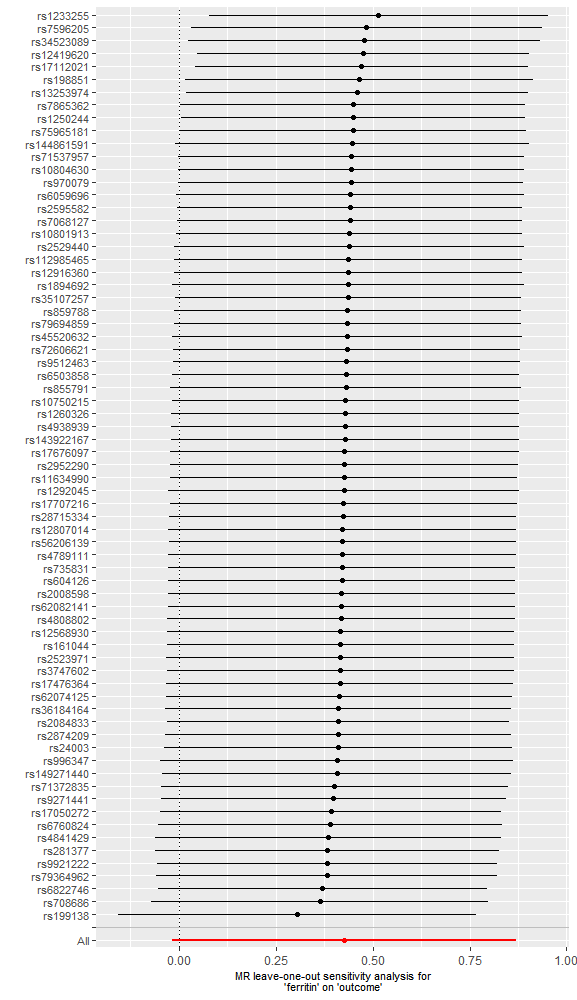


**Figure S10: Leave-one-out test plot of the causal association between serum iron and postmenopausal osteoporotic fracture**


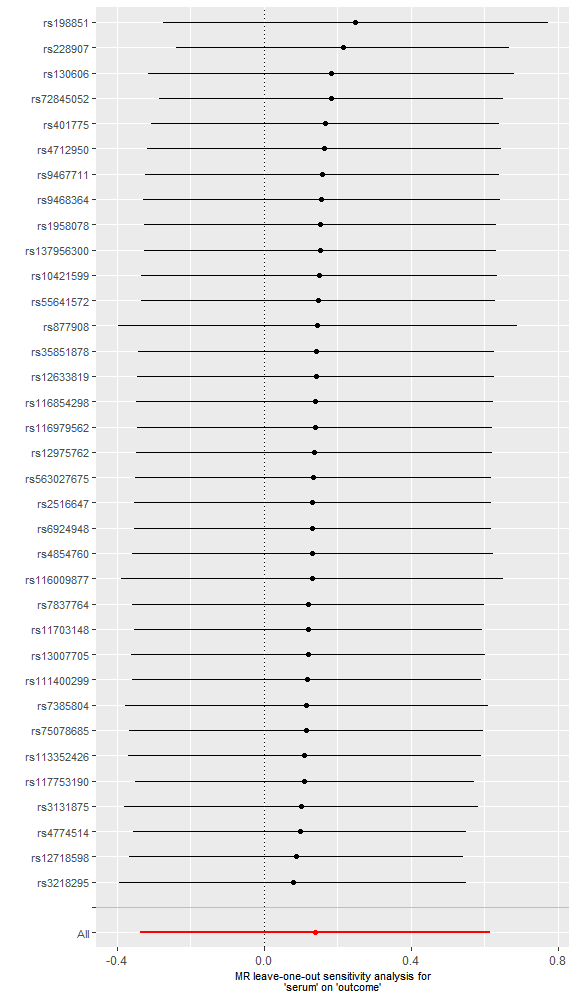


**Figure S11: Leave-one-out test plot of the causal association between total iron-binding capacity and postmenopausal osteoporotic fracture**


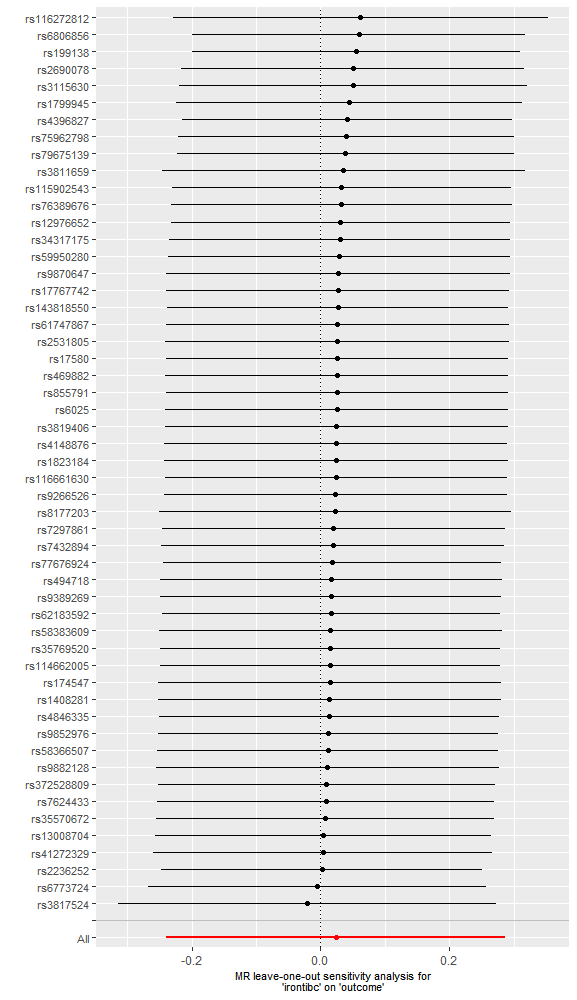


**Figure S12: Leave-one-out test plot of the causal association between transferrin saturation and postmenopausal osteoporotic fracture**


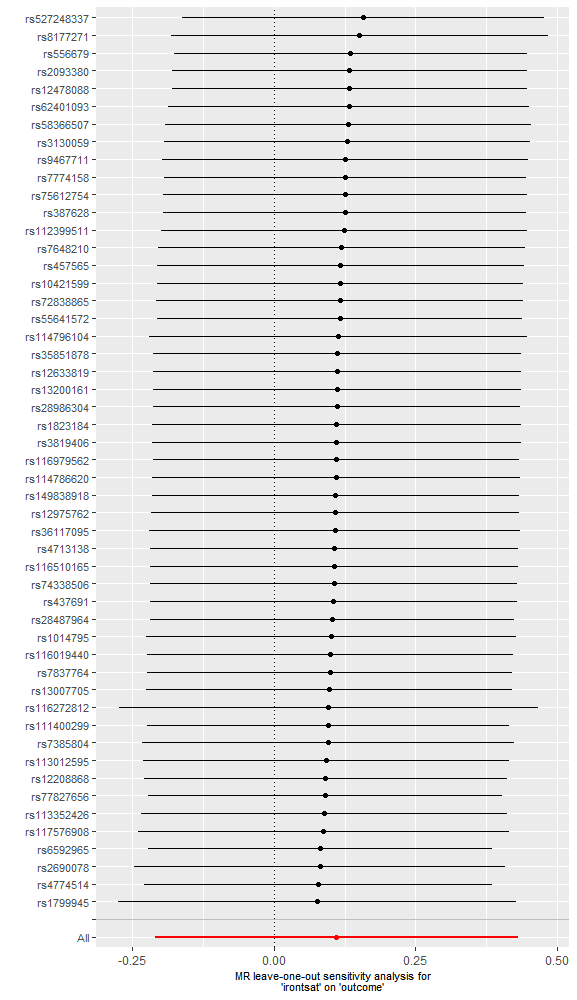

Supplement: Supplementary file 1 [file DataSheet_1.docx]
